# Improving respectful maternity care through group antenatal care: findings from a cluster randomized controlled trial

**DOI:** 10.21203/rs.3.rs-3682833/v1

**Published:** 2023-12-18

**Authors:** Theresa Norpeli Lanyo, Ruth Zielinski, Vida A. Kukula, Veronica E.A. Apetorgbor, Bidisha Ghosh, Nancy A. Lockhart, Jody R. Lori

**Affiliations:** University of Michigan–Ann Arbor; University of Michigan–Ann Arbor; Dodowa Health Research Centre; Dodowa Health Research Centre; University of Michigan–Ann Arbor; University of Michigan–Ann Arbor; University of Michigan–Ann Arbor

## Abstract

**Background:**

Disrespect and patient mistreatment are identified as barriers to care-seeking and low uptake of facility-based deliveries. These mitigating factors have led to slow progress in the achievement of maternal and child health targets, especially in Ghana. Group antenatal care, as an alternative to individual antenatal was implemented to explore the impact on outcomes, including mothers’ perception of respectful care.

**Methods:**

A cluster randomized controlled trial was conducted in 14 health facilities across four districts in the Easter Region of Ghana. These facilities were randomized to intervention or control using a matched pair. Data was collected at several timepoints: enrollment (Time 0), 34 weeks gestation to 3 weeks post-delivery (Time 1), 6 to 12 weeks post-delivery (Time 2), 5 to 8 months post-delivery (Time 3), and 11 to 14 months post-delivery (Time 4). Questions related to respectful care were asked at Time 2, while a focus group discussion (FGDs) was conducted as part of a process evaluation to examine participants’ experiences about respectful maternity care.

**Results:**

The findings from the intervention group indicate that participants perceived higher levels of respect in comparison to the control group. Privacy and con dentiality were maintained. They believed they had been provided with adequate information, education, and counseling, empowering them to make informed decisions. Participants perceived a shortened waiting time and reduced discrimination in care provision. Generally, there were higher levels of satisfaction with antenatal care.

**Conclusion:**

The provision of respectful maternity care, which is essential to increasing healthcare utilization, has been demonstrated to correlate positively with group antenatal care.

## Introduction

### Background

The World Health Organization (WHO) identifies respectful maternity care (RMC) as a key component of patient-centered care, focusing on the individuality of women 2. This care respects dignity, privacy, and confidentiality, protects from injury and maltreatment, and allows for informed decision-making with continuous support throughout labor and delivery (1).

Providing high-quality, respectful, compassionate, and evidence-based care is critical for the well-being of every mother and family (2, 3). This approach to care ultimately contributes to the positive experience envisioned by all women (4–6).

Despite global health efforts to improve maternal and newborn outcomes, maternal and child mortality remains a significant health problem. Nearly 287,000 women die annually from preventable causes worldwide, and almost 95% of these global deaths occur in low and middle-income countries (LMICS), with Sub-Saharan Africa (SSA) accounting for 70% (7). The Sustainable Development Goal (SDG) #3 targets the reduction of the global maternal mortality ratio (MMR) to less than 70 per 100,000 births by 2030 (8). However, strategies to improve access, utilization, and increase skilled birth attendance (9) continue to encounter many challenges.

Research shows women face various forms of disrespect and mistreatment throughout pregnancy and delivery (10–12), including physical, psychological, verbal, or sexual abuse, stigma, discrimination, detention in institutions, extortion, lack of supportive care, insufficient communication, and low care standards (13–15).

An unfortunate consequence of disrespectful care is decreased access to and usage of healthcare services (16). This hampers the progress toward reducing MMR, particularly in poor and LMICs (17–21) by reducing the likelihood of women using facility-based maternity care(10, 18, 22).

In Ghana, though skilled birth attendance rates have steadily increased over the years (23, 24), the lack of respectful treatment in healthcare facilities has been identi ed as a signi cant impediment to healthcare usage (17, 25–28). It is essential to evaluate care delivery strategies, as RMC plays a critical role in attaining care adherence among pregnant women and generally improves healthcare utilization for meeting global targets (9, 16, 29–31).

Predictably, threats to public health and human rights have prompted the current recognition and global discourse surrounding RMC. In 2015, WHO issued a global mandate urging concerted efforts worldwide to eliminate disrespect and abuse during facility-based care (32). The emphasis is to consistently and critically assess and evaluate the quality of care offered to pregnant women (33). This extends to the standard of care and the ethical conduct of midwifery practice (19, 20, 34, 35).

The White Ribbon Alliance (WRA) initiated seven rights for childbearing women to foster RMC. These are principles grounded on dignity, con dentiality, autonomy, respect, equity, culturally sensitive communication, information sharing, and shared decision-making void of ill-treatment, stigma, and discrimination (36, 37). It also aims at identifying strategies to enhance respectful care at every patient-provider interaction (34, 38).

Although a standardized approach to measuring mistreatment is still evolving (29), scholarly investigations have broadened our understanding of quality RMC. These studies have revealed that RMC encompasses more than the mere absence of mistreatment (19).

### Group Antenatal Care

Group antenatal care (G-ANC) uses objective clinical evaluation and treatment, nonhierarchical participatory learning, and peer support to offer context-specfic care for women (39). This behavior-based support model promotes patient involvement through health literacy and self-efficacy (40). While standard ANC emphasizes provider-patient interaction to raise women’s awareness, G-ANC presents health information in a culturally appropriate way to create group strength. Based on the *WHO Recommendations on Antenatal Care for a Positive Pregnancy Experience* (6), G-ANC places women at the center of service delivery to improve access, engagement, and satisfaction.

Developing innovative ways of incorporating RMC into routine antenatal care is paramount. Approaches like the G-ANC model, aimed at enhancing RMC and improving maternal and neonatal health outcomes, can potentially improve and increase facility-based care.

A cluster randomized controlled trial (RCT) of G-ANC was conducted in Ghana with overarching aims to determine the difference between groups in birth preparedness, care seeking, and birth outcomes. The objective of this study is to examine the experience of care between women in the intervention and control groups quantitatively and qualitatively with those participating in G-ANC.

## Methods

### Description of the Intervention

The G-ANC model of care comprised nine meetings; the first ANC visit was an individual session that followed the traditional format of history taking, physical examination, and laboratory testing. Subsequent visits were conducted in a group format, with a midwife as the facilitator, a community health nurse as an assistant, and 10–14 women of similar gestation, followed by eight G-ANC meetings. The pregnant women checked each other’s blood pressure and weights, after which a facilitated discussion was held related to the topic pertinent to that gestational age. For example, early meetings cover topics such as using bed nets and malaria prophylaxis, whereas later meetings covered family planning and newborn care.

### Guiding Framework

The White Ribbon Alliance’s universal rights of Respectful Maternity Care for childbearing women was used as a guiding framework. These rights include 1) freedom from harm and ill-treatment, 2) right to information, informed consent and refusal, and respect for choices and preferences, including companionship during maternity care, 3) confidentiality and privacy, 4) dignity, respect, 5) equality, freedom from discrimination, equitable care, 6) right to timely healthcare and to the highest attainable level of health, and 7) liberty, autonomy, self-determination, and freedom from coercion (41). This seminal work was undertaken to focus on the interpersonal aspects of care: physical abuse, non-consented care, non-confidential care, non-dignified care (including verbal), discrimination based on specific attributes, abandonment or denial of care and detention in health facilities (42).

### Study Design and Setting

A cluster RCT was conducted in 14 health facilities across four districts in the Eastern Region of Ghana. These facilities were randomized to intervention or control using matched pairs. Matching and randomization was completed using nbpM matching package from R software (version 1.5.0) (40). Facility locations were strategically distant to prevent cross-group contamination. Within each facility pair, one was randomly assigned to the intervention group (G-ANC), while the other served as the control group, receiving standard, individual care. Details of the predefined primary and secondary outcome measures can be accessed at ClinicalTrials.gov on 25/07/2019. The RCT: NCT04033003. Comprehensive details of the methods are reported elsewhere (40).

#### Sample.

Sample size was determined by change in birth-preparedness, complication readiness index-scores, and the percent change in babies obtaining postnatal checkups within the first two days after birth and women obtaining maternal postpartum check-ups (40).

Recruitment into the study took place at the individual health facilities, with research staff collaborating closely with clinic personnel to identify eligible women attending their first ANC visit. Eligibility criteria included gestation less than 20 weeks, proficiency in Dangme, Ga, Aka, Ewe, or English language, aged over 15, and with lower-risk pregnancy. Women were introduced to the study by the midwife, and if they expressed interest in participating, they were directed to the research assistant for further details. The research assistants explained the study, and those willing to participate were guided through the informed consent procedure (40). Informed consent was obtained from all participants. The consents were orally presented to participants and witnessed by a healthcare personnel. The participant appends a signature or places a thumbprint after all clarifications have been provided.

Focus group discussions were conducted as part of a process evaluation of group ANC participants each of the seven sites included in the RCT. Details on the qualitative data collection methodology reported elsewhere (43). Led by a research team member, one or two focus groups, were conducted each intervention site with women in the intervention group. The focus groups were conducted in a private setting within the clinical area after informed consent was given. The discussion was done following the final G-ANC meeting which were voluntary.

### Data Collection

Recruitment of participants began in July 2019 with target recruitment reached in November 2021. Data collection ended in June 2023. Quantitative data collection occurred at several timepoints: enrollment (Time 0), third trimester (Time 1), 6 weeks post birth (Time 2), 6 months’ post birth (Time 3), and one year post birth (Time 4). Demographic data was collected at baseline (Time 0) and respectful maternity care questions were collected at Time 2. Participants were lost or excluded after randomization due to miscarriage, if they did not G-ANC sessions, expressed they were no longer interested, or inability to contact the participant.

Twenty-one RMC items were developed and asked of all study participants at 6 weeks’ post-birth. This timepoint was selected to maximize participant recall of care received. Items encompassed freedom from harm and ill-treatment, information received, dignity and respect, and the right to quality care that is equitable. Questions were dichotomous responses as “yes” or “no.”

All data were collected in the local language following informed consent. Quantitative data were recorded by trained research assistants (RAs) on encrypted, password-protected tablets. Qualitative data were collected through focus group discussions where participants were encouraged to share their perceptions of group care. All focus groups were audiotaped, translated, and transcribed verbatim.

### Data Analysis

For the demographic data, categorical variables were compared using the chi-square test. Maternal age and wealth index were analyzed using a 2-sample t-test, and the number of previous pregnancies was tested using the non-parametric Mann-Whitney Wilcoxon test. For the 21 categorical items related to RMC, chi-square test was used to compare the outcome between the G-ANC (intervention) and individual (control) groups. Results for each comparison are reported individually as percentages with signi cance set at a p-value less than 0.05.

Content analysis was employed for the qualitative data using the pre-defined universal rights described by the White Ribbon Alliance (41). The analysis of transcripts was conducted using the following steps: (a) transcripts were read by four authors (TNL, JRL, RZ, NL) for general impressions; (b) participant quotes were sorted into preliminary categories guided by the WRA universal rights by two authors (JRL, TNL); (c) coding was double-checked by the two additional authors (RZ, NL) and any discrepancies were resolved; and (d) consensus on the sorted data was achieved by all authors.

## Results

A total of 1,761 participants were enrolled in the study, organized into 12 groups comprising 10–14 women of similar gestational age. Most participants were under age 35 (84%), and a significant proportion were either married, cohabitating, or living with their significant other (96%). Most had previously given birth, with only 20% of participants pregnant for the first time (Table 1).

Overall, the majority of the women receiving maternity care in this study reported experiencing RMC, as evidenced by nearly all respondents indicating that the midwife called them by name and treated them respectfully (Table 2). One exception was the item “Would you say you were treated differently because of any personal attribute like your age, marital status, number of children, education, or wealth? In the control group, 17% of women responded “yes” whereas in the intervention group, only 7.5% responded yes (p < 0.0001).

Women in both groups received comprehensive care, including being weighed, blood pressure taken, and having tetanus and antimalarial prophylaxis. However, there were significant differences in the intervention group in several of the items related to the right to quality care. For example, women in the G-ANC experienced less waiting time (92% in G-ANC said the wait time was okay vs. 81% in the control group, p < 0.0001). With respect to information, 95% of women in G-ANC reported being told what to expect during the pregnancy versus 87% in the control group (p < .0001). Similarly, they were significantly more likely to be counseled on what to eat (group ANC 99.7% vs. control 94%, p < .0001), breastfeeding (97% vs. 81%), the purpose of tests (group ANC 90% vs. control 82% p < .0001) and medicines given (group ANC 96% vs. control 89%). There was a high level of satisfaction with the ANC received in both groups (99% in group ANC vs. 98% in control).

Ten focused groups s were conducted with 92 participants (5–13 women per group) after the final group ANC meetings. One focus group was conducted at four intervention sites, and two focus groups were conducted at three of the sites. Focus groups lasted approximately 30 minutes. Several women in the intervention groups had delivered and continued attending with their newborns.

### 1. Freedom from harm and ill-treatment

Freedom from harm and ill-treatment, according to the WRA’s *Respectful Maternity Care Charter* (41), is not limited to the absence of physical violence but rather encompasses that women should be cared for in a compassionate and gentle way.

Women in the focus groups talked about feeling cared for. One participant noted,

“*Me too am very happy with the midwife because she pampers and cares for everyone in a friendly manner, and so that is what I am happy about*.”

Another woman stated,

“*So, this is why I am happy, she takes care of us*.”

And yet another described how the process differed from her earlier experience,

“*Am back again, my previous delivery was here but this particular delivery is exceptional. I have never come across midwives like these. But for this I feel they already knew us*.”

### 2. Right to information, informed consent and refusal, and respect for choices and preferences, including companionship during maternity care.

The RMC Charter is explicit in a woman’s right to information, identifying that every woman has the right to receive information, refuse care, have autonomy, and be provided with informed consent.

Participants stressed appreciation for information sharing.

“*This will be my fifth child. There are many things I do not know about the previous four pregnancies…but now with this group antenatal care, I know what is normal and what is not. It has really help me remember and learn a lot*.”

Another woman stated,

“*Concerning checking our BP. We didn’t know the benefits of doing it but during our antenatal visit and teaching during antenatal help us to know the benefits of checking our BP. Maybe when we come and they check, we think it is the doctor’s work that they do every day, but we don’t know why they check. But the teaching helped us know the benefits of it*.”

### 3. Confidentiality and privacy

Confidentiality and privacy are discussed at the first group visits by the midwife leading the group ANC visit. It is explained to participants that what is shared by participants in the group visits is confidential and should not be discussed outside the group setting of ANC.

While confidentiality and privacy were not explicitly addressed in the focused groups by the participants, they reported feeling comfortable in a group setting. One participant noted,

“*Am happy about how we are in the group because we are all free to talk to each other*.”

Another stated:

“*You are able to have good communication with the nurses*”.

### 4. Dignity and respect

Dignity and respect address the right of the childbearing woman to be treated with compassion and respect. Verbal abuse and humiliation are not to be tolerated in respectful maternity care.

Participants expressed their happiness with the way they were treated during group ANC visits. A woman experiencing her fourth pregnancy noted,

“*When this one is added I have given birth to four. But how we come to meeting and how they [midwives] treat us and comparing to the three that I delivered [previously], it was not so because they have love for us, they have patience for us…I hurry up to come*.”

Another woman said:

“*Am happy about how when we come that even when you tell them about what is wrong with you, they have patience to take care of you.” Yet another woman stated, “Me, I am happy about how friendly they [midwives] are with us when we come and even when we are late, they respect us and everything*.”

### 5. Equality, freedom from discrimination, and equitable care

The fifth right within the RMC Charter identifies that no one is allowed to discriminate against a woman or her newborn because of something they think or do not like about either one.

This participant noted,

“*I have two children, one of them is 3 years old and the other is 1 and a half and I’m also pregnant. There are places that when you go they will be shouting at you that don’t bring the children there. Sometimes when you come [here], they can even hold the child for you so that they can take care of you even when they are palpating the stomach. So even when you are home, you are in a hurry that it will be time so that you can come and learn*.”

### 6. Right to timely healthcare and to the highest attainable level of health

Women and their newborns are entitled to the highest quality of care attainable according to the RMC Charter. This includes being paid attention to when a problem is identified and treated in a timely manner.

One participant said,

“*How the midwives handle us makes us comfortable to tell them when we have a problem*.”

Yet another woman declared,

“*And if there is something wrong and you call her, she will talk to you in a calm way and clarify things*.”

Compared to her previous care, this participant said,

“*Am back again, my previous delivery was here but this particular delivery is exceptional. I have never come across midwives like these, but for this I feel they already knew us*.”

### 7. Liberty, autonomy, self-determination, and freedom from coercion

Regarding autonomy and self-determination, women in the focus groups were clear on how group care supported them in this respect. One woman said,

“*I am able to express my views.” Another woman noted, “It has taken away the fear we used to have when pregnant*.”

Additionally, one woman talked about how group antenatal care gave her the confidence to speak to her husband about saving money for the delivery,

“*I was somebody that keeping money was very di cult for me. So, most of the time when I save and there is something to be done the man [husband] will ask me ‘oh maame, please don’t you have this’. So, I remember one day I told him ‘I beg see it has been written here that we should be saving money so don’t be collecting the money…so I told him he should allow me to save something small for myself*.”

## Discussion

Addressing preventable maternal and newborn mortality, as well as improving the overall health and well-being of women and children, is a global concern. Following WHO’s quality of care framework for pregnant women (6), G-ANC places women at the center of service delivery (44) to decrease waiting time, improve patient-provider communication, and address the seven rights of childbearing women (37) to tackle disrespect and abuse.

Triangulating the qualitative and quantitative data results strengthens the findings of participants’ perspectives of the intervention on RMC per the universal rights of the White Ribbon Alliance (41). The majority of women in both individual and group antenatal care indicated that the care provided by the midwives in Ghana was respectful. However, the perceived care of women in G-ANC was more positive, as measured by the 21 items related to RMC (Table 2), than that of the control group.

Participants in the focused groups clearly articulated they were being supported and cared for in a compassionate and caring way by the midwives during G-ANC. Respectful maternity care was demonstrated in all seven of the universal rights of Respectful Maternity Care for childbearing women. Participants reported that their physical, emotional, and psychological needs were being addressed through G-ANC.

The findings of connectedness and mutual respect among healthcare providers and patients are consistent with similar G-ANC reports where patients feel safe, warm, and respected (45). Self-expression with a sense of privacy and con dentiality, dignified treatment with no evidence of discrimination, having the best quality of care, and the freedom to make informed decisions were key findings.

Respect is a basic human right essential to professionalism and ethical conduct in maternal and child health (46). Miller and colleagues believe that the significance of evidence-based care falls short when it lacks elements of humanity, dignity, and respect in its delivery (3). Giving women a voice in their care is crucial to ensuring they are active participants rather than passive users (47). This is a key component of the WHO/UNICEF petition for quality, equity, and dignity (48)

While disrespect and mistreatment have been observed around the world and have become a global health problem, our findings show that women enrolled in G-ANC report improved communication with their midwives and satisfaction with the interpersonal aspects of care provided during their ANC visits, which supports previous findings from various interventions studies of group ANC carried out in LMICs, Rwanda (45, 49), Nigeria (50) and Kenya (51). Our findings contribute to the evidence for providing RMC, as recommended by Vogel et al. (2016), and to ultimately achieve global objectives of reducing maternal and child mortality through behavioral-based support (6, 9, 32, 34, 52).

There are several limitations to this research. Focus groups were conducted with only the women participating in G-ANC. This study utilized self-reported quantitative data, which may introduce social desirability bias. The items related to RMC have not undergone psychometric testing of validity and reliability. Additionally, this study was limited to the Eastern Region of Ghana, reducing generalizability. However, examining the combination of qualitative and quantitative data using the White Ribbon Alliance’s (WRA) universal rights of Respectful Maternity Care for childbearing women as a guiding framework ensures that the strength of the other balances the limitations of one type of data collection.

## Conclusion

The G-ANC model has the potential to improve RMC, starting with ANC visits. G-ANC encourages a meaningful and therapeutic relationship between the provider and patient. It detects and tackles direct and indirect individual and contextual barriers while providing respectful care from the patient’s perspective. This is vital for promoting behavioral changes in the patient to increase healthcare use.

This paper adds to the growing body of literature on the use of G-ANC models to achieve RMC and reduce inequities by maintaining dignity, privacy, and confidentiality, freedom from harm and mistreatment, and informed choice for women during antenatal care.

## Figures and Tables

**Table 1:** Participant Demographics

Categorical Variables	Total (N = 1761)	Control (n = 884)	Intervention (n = 877)	p-value
**Age**				
<25	501 (28%)	266 (53%)	235 (47%)	0.193
25–34	987 (56%)	477 (48%)	510 (52%)	
35 or older	273 (16%)	141 (52%)	132 (48%)	
**Education**				
Primary	246 (14%)	120 (49%)	126 (51%)	0.6895
Middle School or less	829 (49%)	429 (52%)	400 (48%)	
Secondary	459 (27%)	223 (49%)	236 (51%)	
Tertiary	164 (10%)	83 (51%)	81 (49%)	
**Partner Education**				
Middle School or less	666 (39%)	335 (50%)	331 (50%)	0.8525
Secondary	627 (37%)	306 (49%)	321 (51%)	
Tertiary	261 (16%)	126 (48%)	135 (52%)	
N/A, Unknown	137 (8%)	64 (47%)	73 (53%)	
**Religion**				
Christianity	1646 (93%)	835 (50.4%)	811 (49.6%)	0.1185
Muslim	97 (6%)	39 (39%)	58 (61%)	
Other	18 (1%)	10 (56%)	8 (44%)	
**First Pregnancy**				
No	1412 (80%)	703 (50%)	709 (50%)	0.4876
Yes	349 (20%)	181 (52%)	168 (48%)	
**Location of Delivery**				
Hospital/Polyclinic/Health Center	1711 (97%)	853 (50%)	858 (50%)	0.0904
**Age**				
Other	50 (3%)	31 (62%)	19 (38%)	
**Continuous Variables Mean (SD)**				
Maternal age	28.2 (5.8)	28.1 (6)	28.3 (5.6)	0.5042
Wealth index	6.8 (2.4)	6.9 (2.4)	6.9 (2.3)	0.6174
Number of previous pregnancies	3.5 (1.4)	3.5 (1.4)	3.5 (1.5)	0.7075

**Table 2:** Respectful Maternity Care

	Total	Control	G-ANC Intervention	p-value
***How did you feel about the amount of time you waited before being attended to?***				
It was okay	1015 (86%)	479 (81%)	536 (92%)	<0.0001
It was too long	160 (14%)	111 (19%)	49 (8%)	
*Missing/don’t remember*	6			
**Did the midwife introduce herself to you?**				
Yes	806 (69%)	315 (53%)	491 (84%)	<0.0001
No	368 (31%)	275 (47%)	93 (16%)	
*Missing/don’t remember*	7			
**Did the midwife call you by name?**				
Yes	1161 (99%)	581 (98.5%)	580 (99%)	0.348 NS
No	14 (1%)	9 (1.5%)	5 (1%)	
*Missing/don’t remember*	6			
**Were you weighed?**				
Yes	1170 (99%)	589 (99%)	581 (99%)	0.6929 NS
No	7 (1%)	3 (1%)	4 (1%)	
*Missing/don’t remember*	4			
**Was your blood pressure taken?**				
Yes	1168 (99%)	587 (99%)	581 (99%)	0.7515 NS
No	9 (1%)	5 (1%)	4 (1%)	
*Missing/don’t remember*	4			
**Were you given a tetanus injection?**				
Yes	1123 (96%)	560 (96%)	563 (97%)	0.2870 NS
No	43 (4%)	25 (4%)	18 (3%)	
*Missing/don’t remember*	15			
**Were you given malaria medication?**				
Yes	1109 (94%)	564 (95%)	545 (93%)	0.1495 NS
***How did you feel about the amount of time you waited before being attended to?***				
No	67 (6%)	28 (5%)	39 (7%)	
*Missing/don’t remember*	5			
**Were you told where to go if you had any complications?**				
Yes	1107 (96%)	545 (94%)	562 (97%)	0.0048
No	52 (4%)	36 (6%)	16 (3%)	
*Missing/don’t remember*	21			
**Were you told what to expect during your pregnancy and delivery?**				
Yes	1067 (91%)	510 (87%)	557 (95%)	<0.0001
No	106 (9%)	79 (13%)	27 (5%)	
*Missing/don’t remember*	8			
**Did the midwife ever talk to you about planning how to get to the health facility?**				
Yes	1092 (93%)	514 (87%)	578 (99%)	<0.0001
No	81 (7%)	75 (13%)	6 (1%)	
*Missing/don’t remember*	8			
**Did the midwife ever talk to you about what to eat?**				
Yes	1136 (97%)	554 (94%)	582 (99.7%)	<0.0001
No	38 (3%)	36 (6%)	2 (.3%)	
*Missing/don’t remember*	8			
**Were you given any information or counseled about breastfeeding?**				
Yes	1047 (89%)	479 (81%)	568 (97%)	<0.0001
No	126 (11%)	111 (19%)	15 (3%)	
*Missing/don’t remember*	8			
Did you feel the midwife treated you with respect?				
Yes	1165 (99%)	585 (99%)	580 (99%)	0.4876 NS
No	8 (1%)	5 (1%)	3 (1%)	
*Missing/don’t remember*	8			
**Did you feel the midwife treated you in a friendly manner?**				
***How did you feel about the amount of time you waited before being attended to?***				
Yes	1148 (98%)	568 (96%)	580 (99%)	0.0002
No	27 (2%)	23 (4%)	4 (1%)	
*Missing/don’t remember*	6			
**Did you feel you understood the purpose of tests you were asked to do?**				
It was okay	1010 (86%)	482 (82%)	528 (90%)	
It was too long	165 (14%)	109 (18%)	56 (10%)	
*Missing/don’t remember*	6			
**Did you feel you understood the purpose of medicines you were given?**				
Yes	1083 (92%)	524 (89%)	559 (96%)	<0.0001
No	92 (8%)	67 (11%)	25 (4%)	
*Missing/don’t remember*	6			
**Did you feel you could have asked the midwife any questions you had?**				
Yes	1095 (93%)	544 (92%)	551 (94%)	0.1173 NS
No	80 (7%)	47 (8%)	33 (6%)	
*Missing/don’t remember*	6			
**Did the midwife ask you if you had questions?**				
Yes	1028 (88%)	477 (81%)	551 (95%)	<0.0001
No	143 (12%)	113 (19%)	30 (5%)	
*Missing/don’t remember*	10			
**Did the midwife ask you for money?**				
Yes	64 (5%)	31 (5%)	33 (6%)	0.7648 NS
No	1110 (95%)	559 (95%)	551 (94%)	
*Missing/don’t remember*	8			
**Were you treated differently because of any personal attributes like your age, marital status, number of children, your education, or wealth?**				
Yes	144 (13%)	100 (17%)	44 (8%)	<0.0001
No	1019 (87%)	486 (83%)	533 (92%)	
***How did you feel about the amount of time you waited before being attended to?***				
*Missing/don’t remember*	17			
**How satisfied were you with the services received during your antenatal care?**				
Satisfied	1159 (99%)	579 (98%)	580 (99%)	0.05 NS
Not Satisfied	16 (1%)	12 (2%)	4 (1%)	
*Missing/don’t remember*	6			

## Data Availability

The datasets generated and/or analyzed during the current study are available in the Deep Blue repository, https://deepblue.lib.umich.edu/data

## Abbreviations

ANC: Antenatal Care
FGD: Focus group discussions
G-ANC: Group Antenatal Care
LMICs: Low and middle-income countries
RA: Research Assistant
RMC: Respectful Maternity Care
SDG: Sustainable Development Goal
SSA: Sub-Saharan Africa
WHO: World Health Organization
WRA: White Ribbon Alliance
